# Pre-hospital blood transfusion – an ESA survey of European practice

**DOI:** 10.1186/s13049-020-00774-1

**Published:** 2020-08-14

**Authors:** Karl-Christian Thies, Anatolij Truhlář, Damian Keene, Jochen Hinkelbein, Kurt Rützler, Luca Brazzi, Benoît Vivien

**Affiliations:** 1 Dept of Anaesthesiology, Critical Care and Pain Medicine, Greifswald University Medical Centre, Greifswald, Germany; 2 Emergency Medical Services of the Hradec Kralove Region, Hradec Kralove, Czech Republic; 3 Department of Anaesthesiology and Intensive Care, Charles University in Prague, Faculty of Medicine, Hradec Kralove, Czech Republic; 4 University Hospital Hradec Kralove, Hradec Kralove, Czech Republic; 5 Department of Military Anaesthetics and Critical Care, Royal Centre for Defence Medicine, Birmingham, United Kingdom; 6 Department of Anaesthesiology and Intensive Care Medicine, Medical Faculty and University Hospital of Cologne, Cologne, Germany; 7 Departments of General Anaesthesiology and Outcomes Research, Cleveland Clinic, Anaesthesiology Institute, Cleveland, USA; 8 Department of Surgical Sciences, University of Turin, Turin, Italy; 9 Italy Department of Anesthesia, Intensive Care and Emergency, Città della Salute e della Scienza’ hospital, Turin, Italy; 10 University of Torino, Turin, Italy; 11 SAMU de Paris, Anaesthesiology and Critical Care Department, Universitary Hospital Necker - Enfants Malades, APHP Centre - University of Paris, Paris, France

**Keywords:** Prehospital care, Emergency medical services, Transfusion, Trauma, Treatment, Europe

## Abstract

**Background:**

Blood products are a lifesaving commodity in the treatment of major trauma.

Although there is little evidence for use of pre-hospital blood products (PHBP) in seriously injured patients, an increasing number of emergency medical services have started using PHBP for treatment of major haemorrhage.

The primary aim of this survey was to establish the degree of prehospital blood product use throughout Europe and discover main indications. The secondary aim was to evaluate opinions about PHBP and also the experience and the personal views of its users.

**Methods:**

The subcommittee for Critical Emergency Medicine of the European Society of Anaesthesiology (ESA) held an online survey of European Helicopter Emergency Services (HEMS) and all French Services d’Aide Médicale Urgente (SAMU) regions. It contained 13 questions both open and multiple-choice about the frequency transfusions are carried out, the PHBP used and the perceived benefit. The survey was distributed to the corresponding HEMS leads in 14 European countries.

**Results:**

In total there were 172 valid responses; overall 48% of all respondents have prehospital access to packed red cells, 22% to fresh plasma and 14% use lyophilised plasma. Besides blood product administration, 94% of all services use tranexamic acid. Sixty five percent of all replies came from French and from German services (37 and 28% respectively). PHBP were mainly used for trauma related emergencies. France has the highest uptake of use of blood products at 89%, whereas the rate in Germany was far lower at 6%.

Fifty five percent of the service leads felt that PHBP are beneficial, and even lifesaving in individual cases despite being needed infrequently.

**Conclusions:**

We found remarkable dissimilarities in practice between the different European countries. Even if there is not an absolute consensus amongst providers on the benefit of PHBP, the majority feel they are beneficial. The difference in practice is possibly related to the perceived lack of evidence on prehospital blood transfusion. We suggest to include the use of PHBP in trauma registries in order to consolidate the existing evidence.

## Background

Massive haemorrhage is the main cause of preventable death in trauma. The key components of treatment are immediate haemorrhage control and transfusion of blood products to replace volume and manage coagulopathy. The European guidelines on management of major bleeding and coagulopathy following major trauma [1] suggests that bleeding trauma patients should be rapidly transported to major trauma centres to institute appropriate treatment as soon as possible. Despite this, the mortality from exsanguinating injuries is still high. In an attempt to reduce morbidity and mortality, prehospital transfusion of blood products has been initiated in several trauma settings throughout Europe. The benefits are discussed controversially, especially as scientific evidence remains ambiguous.

During the Vietnam War, the US armed forces successfully implemented a pre-hospital blood transfusion (PHBT) program, which was further expanded during subsequent military conflicts. Indeed, the ongoing evolution of military treatment has led to a significant reduction in the mortality from battlefield injuries. PHBT and replacement of clotting factors are part of these modern care bundles, but it is still not clear how far the observed decrease in mortality can be attributed to it [2].

Due to the different injury patterns seen in civilian trauma as well as logistical issues and cost implications, PHBT on a wider scale is introduced only reluctantly in European practice. A recent systematic review of the literature [3, 4] failed to provide evidence to support PHBT. However, this did not include the two recent randomized controlled trials (RCTs), PAMPer (Prehospital Air Medical Plasma) and COMBAT (the Control of Major Bleeding After Trauma Trial). Post hoc analysis combining the two trials suggests there is a benefit when prehospital transfer times are longer than 20 min [5].

### Objectives

In order to identify current European practice and the underlying rationale, the subcommittee for Critical Emergency of the European Society of Anaesthesiology has conducted a survey of advanced pre-hospital care services in multiple European countries on the use of PHBT. The aim was to establish the degree of blood product use, the indications for it and to evaluate opinions of practitioners about using pre-hospital blood products.

## Method

We carried out an online survey using Survey Monkey (San Mateo, CA, USA) software. The clinical leads of all Helicopter Emergency Medical Services (HEMS), in Germany, Sweden, Norway, Finland, Czech Republic, the United Kingdom, Switzerland and Hungary were approached. The German services were approached through the ‘Allgemeiner Deutscher Automobil Club’ (ADAC) and the DRF Luftrettung (DRF). The Swiss services were contacted through the ‘Schweizer Rettungsflugwacht’ (REGA). In France, the survey was distributed through the National ‘Service d’Aide Médicale Urgente’ (SAMU) to all SAMU districts and also includes ground based advanced Emergency Medical Services (EMS). The remaining services were identified by internet search.

The survey consisted of 13 combined open and multiple-choice questions.

The data collection took place between April 1st, 2016 and March 31st, 2017.

The data processing took place in the UK and the temporary data storage in Ireland. The survey was conducted under UK and Irish regulations in line with the UK Data Protection Act 1998 and the ruling of the European Court of Justice from October 6th, 2015.

## Results

**Fig. 1:**
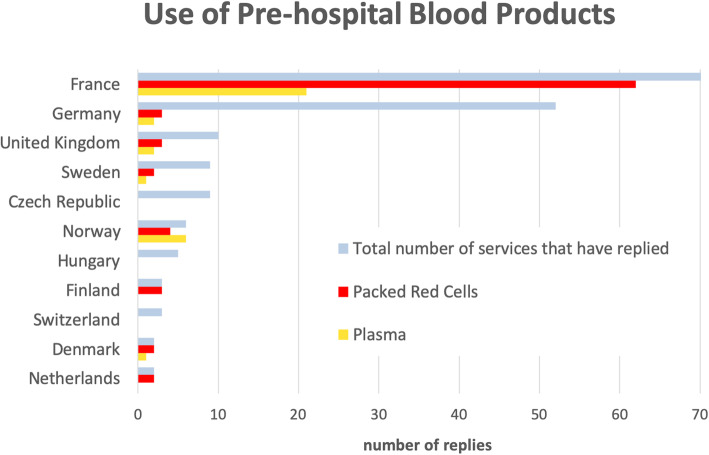
The use of prehospital blood products differs significantly between countries. Packed red cells are mainly used, and some services also use plasma. There is remarkable difference in practice in between France and Germany

The responses cover 189 services from 14 European countries of which 17 were incomplete and excluded. The majority of the replies came from France and Germany (37 and 28% respectively). French services have the highest uptake of use of blood products at 89%, whereas the German rate was far lower at 6% (Fig. 1).

Answers were obtained from 4 out of 4 (100%) HEMS bases in Denmark, 9 out of 10 (90%) HEMS bases in the Czech Republic, 52 out of 65 (80%) HEMS bases in Germany, 70 out of 104 (66%) SAMU districts in France, 7 out of 12 (58%) HEMS bases in Norway, and 2 out of 4 (50%) Dutch HEMS bases.

Overall 48% of all respondents reported to have access to packed red cells, 22% to fresh plasma and 14% use lyophilised plasma. Besides blood product administration, 94% of all services use tranexamic acid in major haemorrhage and two services carry fibrinogen concentrates.

The majority of services that have access to prehospital blood products (PHBP, 77%) use them less than monthly. Only 8% of the services use blood products more than weekly.

**Fig. 2:**
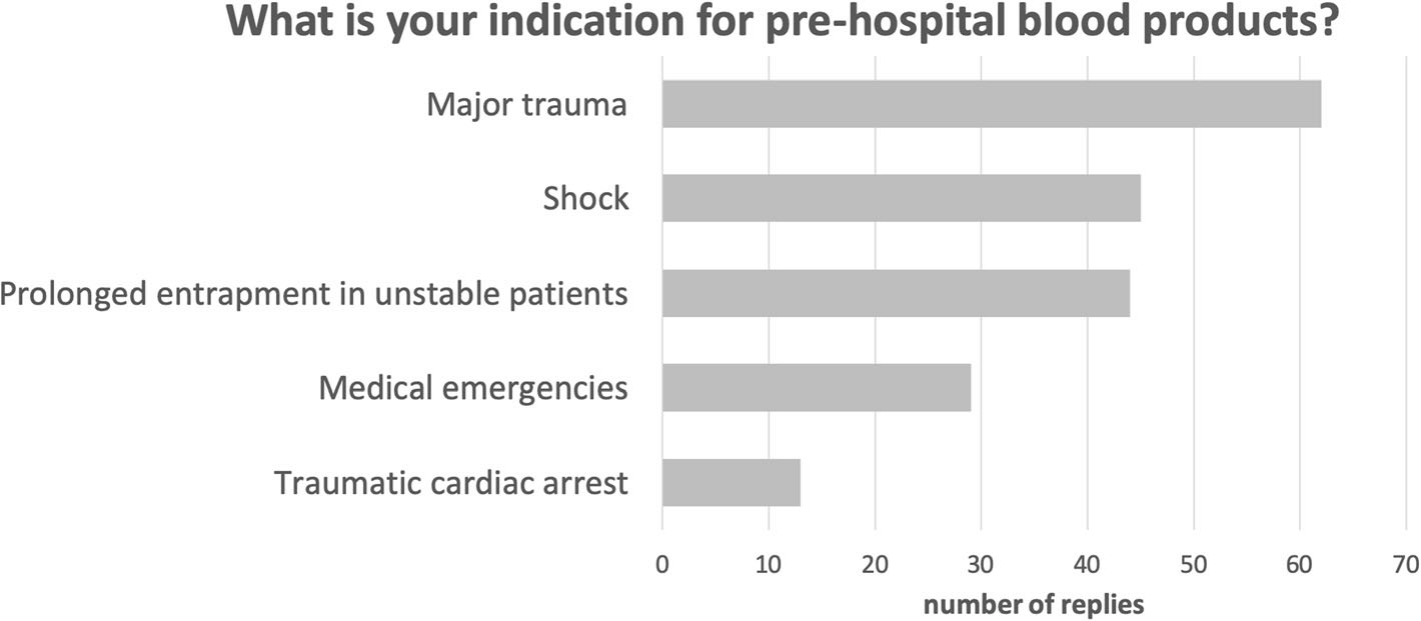
The main indication for prehospital blood transfusion is major trauma

The most common indication for pre-hospital transfusion was major trauma, while medical emergencies play less important role (Fig. 2).

Overall 72% of the respondents have a formal pre-hospital transfusion policy, with only 38% of the services auditing their transfusion practice.

Our results did not indicate any wastage, lost traceability or transfusion reaction. Some services indicated that they have only recently started carrying blood products, with numbers of patients too small to draw any conclusions.

**Fig. 3:**
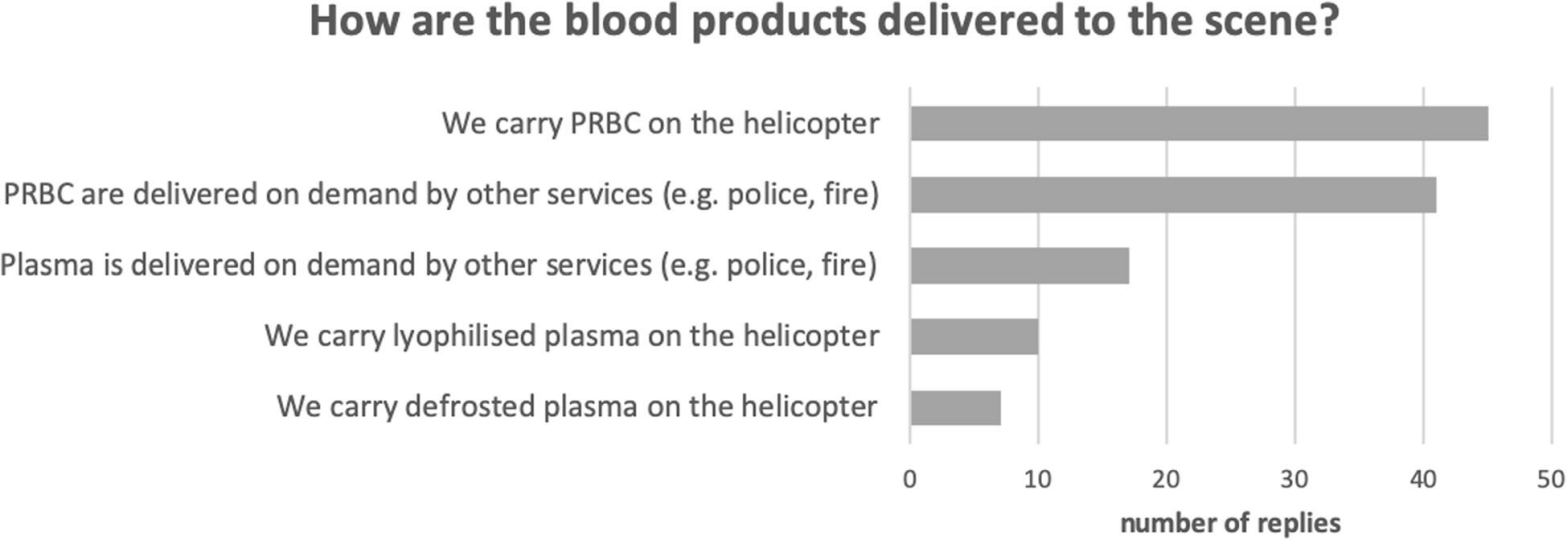
The majority of services carry blood products on the aircraft or on the response vehicle

There are various methods of transporting blood products to the scene of an incident; exactly half of services (50%) carry the blood products on the helicopter or response vehicle. (Fig. 3).

**Fig. 4:**
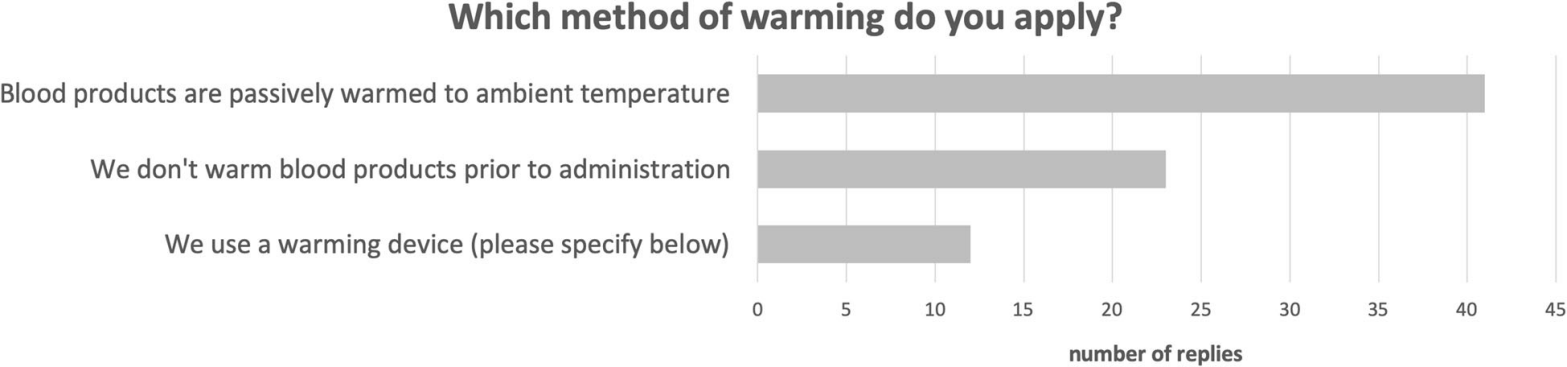
The majority of services do not employ active warming of prehospital blood products prior to transfusion

Active warming of blood products is only performed by 16% of the services. Of the remaining 84, 54% only warm them passively to ambient temperature and 30% do not warm at all (Fig. 4). For active warming, the Belmont® Buddy Lite™ is used by all services apart from one, which employs the QinFlow Warrior (QIF™).

Long transportation times (29%), high prevalence of major trauma (18%) and long access times (18%) to reach patients, were reported as the leading infrastructural of geographical factors that would justify using PHBP.

Two services reported communication problems with the regional Major Trauma Centres that would delay start of massive transfusion on hospital arrival as an infrastructural factor suggesting the use of PHBT. However, 55% of all service leads feel that there are no specific geographical or infrastructural factors in their region suggesting the use PHBT.

**Fig. 5:**
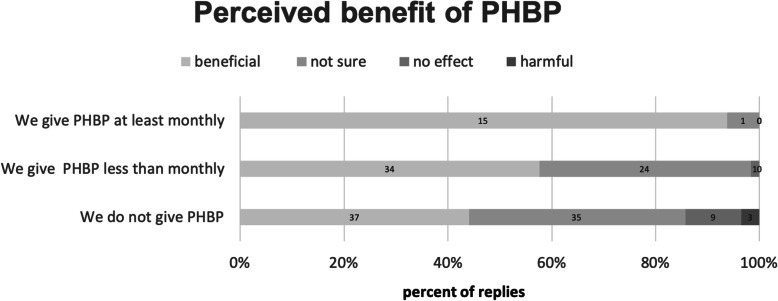
There is no consensus on the beneficial effect of prehospital blood transfusion on patient outcome. Whereas services that use blood products more frequently, are strongly convinced that they are beneficial for their own practice, services that use blood products less frequently or not at all, do not share this view

The views regarding the risks and benefits of PHBT are diverse: 54% of all service leads feel that PHBT is beneficial, 37% are not sure, 6% feel that PHBT does not make a difference, and 3% think that it might be harmful. This perception is higher in those carrying out more PHBT, 15 out of 16 clinical leads amongst the frequently transfusing services regarding PHBT as beneficial (Fig. 5).

The service leads were also asked to give their personal views on their risk/benefit perception of PHBT. In summary, they felt that it was a rarely required intervention, but lifesaving in exsanguinating trauma patients. It was felt that the evidence was weak, that there are no guidelines on the use of PHBP, and that international collaboration is needed to establish the role of blood products in the prehospital field.

**Fig. 6:**
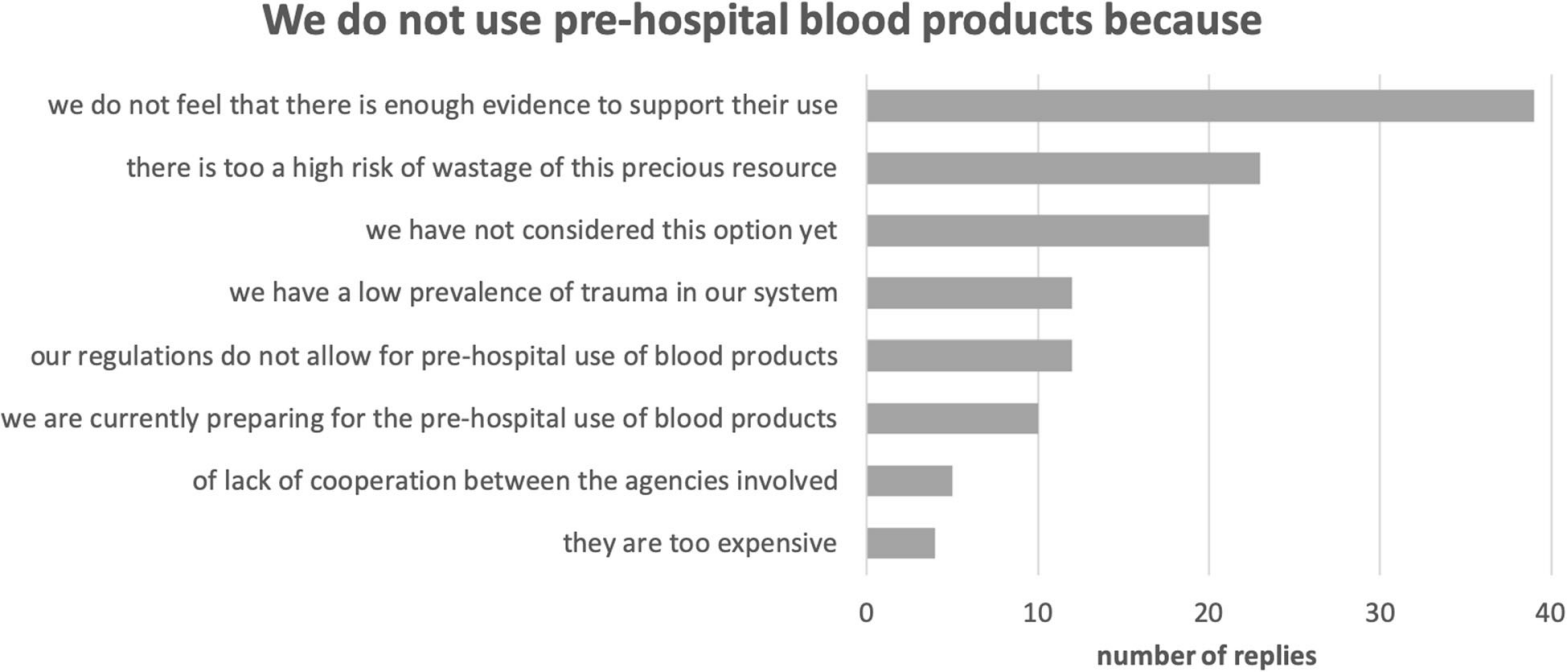
Lack of evidence and risk of wastage are the main reasons not to employ prehospital blood transfusion

There are various reasons for not using PHBT (Fig. 6). The main motives are the perceived lack of scientific evidence and the risk of wastage of blood products. Cost considerations seem to be a less important reason not to use PHBP.

The datasets used and/or analysed during the current study are available from the corresponding author on reasonable request.

## Discussion

Exsanguination is still the major cause of preventable mortality in trauma [6]. Haemorrhage control and haemostatic resuscitation are the cornerstones of in-hospital treatment, with delays in transfusion related to increased mortality [7].

The concept of forward resuscitation has brought blood transfusion to the frontline of armed conflicts [8] and there is little doubt amongst the military community that PHBT has helped to decrease battlefield mortality dramatically over the last 20 years [2].

However, scientific evidence to support PHBT as a standard of care in exsanguinating trauma is weak as the majority of all studies on the topic are either underpowered or retrospective. This has hampered its introduction into civilian practice [4, 9]. Recently two prospective randomized controlled trials have been published providing some evidence regarding the effect of prehospital plasma in trauma patients. Defrosted plasma compared to standard resuscitation is associated with a survival benefit (NNT of 10) for patients undergoing long transports (median transportation time > 40 min) to the receiving hospital (PAMPer) [10]; conversely, in an urban environment, no such benefit could be found (COMBAT) [11]. This may be related to the significantly shorter transportation times in the second study (median transportation time < 20 min) [5]. Of note is that in both studies the mean injury scores (PAMPer: ISS =22, Combat: NISS = 27) were not particularly high, which could be due to low inclusion thresholds or a prevalence of single organ system injury patterns.

A critical appraisal of both studies [12] comes to the conclusion that the administration of plasma to patients undergoing short transportation times is not justified, and that there still is not enough evidence to give firm recommendations on the use of prehospital fresh plasma.

A post hoc analysis of the PAMPer Trial data [13] found a greater survival benefit for patients resuscitated with PRBC and plasma (HR 0.36), instead of plasma (HR 0.68) or PRBC (0.69) compared to crystalloids. However, the PAMPer Trial was not designed to answer this particular question and therefore the results must be read with caution.

Our survey has found a considerable variation in PHBT practice in Europe with the two largest countries France and Germany employing very different approaches. The return rates in both countries were particularly high (85%). In France, 70% of the prehospital services have access to blood products, whereas in Germany PHBT is hardly ever used. In the light of similar trauma rates and comparable patterns of injury in both countries [14], this is a truly surprising finding and we can only speculate on the underlying reasons. One explanation could be that the French SAMU system is mostly hospital-based with the teams departing from emergency departments having easier access to blood products, than the German advanced prehospital care systems that have become detached from hospital services over the last decades. In some services, PHBP need to be picked-up at the emergency departments on request, but they are not accessible to every trauma patient treated on scene. We must also mention, that the French respondents expressed their opinions from perspective of the ground ambulance providers, while in the other countries’ opinions were represented by the HEMS leads treating patients in haemorrhagic shock more frequently. There are also different (Fig. 5) attitudes towards the use of PHBP, which in part could be explained by the absence of robust scientific evidence for or against PHBT, leaving space for opinion-based approaches. These may be driven by individual views and experience, differences in national regulations, and variable regional availability of blood products.

Haemostatic resuscitation in-hospital relies on the use PRBC and plasma. Early administration of plasma aims at maintaining coagulation [15] and mitigating the inflammatory response to trauma as well as the breakdown of the endothelial glycocalyx [16]. Indeed, in-hospitalpatients with exsanguinating haemorrhage would now never be resuscitated with crystalloid based regimes. This is despite there being no RCT that demonstrates its superiority. There are, however, multiple retrospective studies that together have demonstrated its benefits [1]. Currently there are no national or international registries collecting data on PHBP administration making it hard to gain adequate numbers to perform an appropriately powered database analysis.

Risk of wastage of blood products and lack of evidence were given as the main reasons for not using PHBP. Despite this, none of the services using PHBP in our study have reported any wastage. The reported wastage from several other studies is below 2% [3, 17]. Even defrosted plasma, with a short shelf-life of 6 days, can be used in the prehospital environment without wastage. Modern blood boxes can maintain temperatures below six degree Celsius for more than 48 h. This allows for 24 h rotation cycles for blood products between the HEMS base and the corresponding blood transfusion service. In our own services (Greifswald University Medical Center, Germany and Hradec Kralove HEMS, Czech Republic), we have been applying a rotational system for 9/19 months respectively without any wastage. We suppose, that well-established logistics and cooperation between the HEMS and blood transfusion services might overcome this barrier.

It is surprising that 25% of the services do not operate a blood transfusion policy given that it is mandated within EU guidelines [18] for in-hospital use. Equally only 38% of the services audit their transfusion practice. In our view, the management and the governance of a pre-hospital transfusion program must be under the auspices of the regional blood transfusion service to ensure patient safety, traceability of blood products and compliance with the complex regulations around transfusion. This includes a written transfusion policy and a continuous audit.

Blood transfusion bears the risks of transfusion reactions, infections, hypocalcaemia and hypothermia. The unrestrained or prophylactic use of blood products is not justified. A pre-hospital transfusion policy must include clear indications for the use of PHBT and well-defined transfusion triggers to ensure patient safety and a rational use of this precious resource [19]. For the prehospital theatre, it is more difficult to completely define triggers, and other “dynamic” parameters should be considered: rate of bleeding, control of the bleeding, time / distance to the nearest hospital where transfusion could be available, and eventually time for extrication.

Transfusion reactions are not reported in the current study, but a recent systematic review indicates that the incidence of adverse reactions in PHBT is around 1% [20]. This is not surprising because major transfusion reactions (ABO incompatibility) are very unlikely, as blood group O is used for red cell transfusion in major haemorrhagic patients without previous blood group determination. Additionally, the immunological response may be diminished in major trauma due to a temporary suppression of the immune system [21]. It is also likely that minor transfusion reactions go unnoticed in the setting of major trauma resuscitation and massive haemorrhage. The largest prospective study on PHBP, the PAMPer Trial, reported an incidence of 2.2% of minor transfusion related adverse events but no major incidents after transfusion of defrosted FFP. Another recent systematic literature review concludes that the PHBT is safe and that only minor transfusion reactions have been reported so far [3]. The majority of services in our survey do not employ active warming prior to transfusion, which we feel, increases the risk of hypothermia and consequently coagulopathy in major trauma patients.

PHBT is widely used in Europe; and almost 50% of the advanced pre-hospital services (that have responded to our survey) have access to PHBP in the field. Next to PRBC, one third of the services have access to liquid fresh plasma or lyophilized plasma concentrate.

At last, 67 % of the services have expressed their interest in participating on further research into the topic.

### Limitations

- The survey addressed the service leads only and does not consider the views of the complete teams
- The return rates were variable amongst countries. Therefore, results do not represent the prevailing situation in each country but rather individual opinions of prehospital care providers from certain regions.
- Ten of the services that have replied indicated that they were about to start PHBT programmes. It not unlikely that the number of services that currently use PHBT has increased since the data collection, and some services may have introduced new blood products which were not available in the past, e.g. deleucotized whole blood.
- The French SAMU EMS included in the analysis is known to have different organisation compared to the HEMS systems in other countries. Except France, there were no ground ambulance services or their medical directors requested to provide information on use of PHBP in ground services, unless HEMS base was also responsible for operation of physician response vehicles.
- Finally, we must mention that opinions collected were highly subjective, and may have been affected by clinical experience of each respondent, diversity between systems, and incidence of treated patients requiring early transfusion.

## Conclusion

There are considerable national differences with very divergent attitudes and approaches towards PHBT amongst European advanced pre-hospital care services, with little to no consensus amongst the providers on its benefit. However, the services that frequently use PHBT feel it is beneficial.

In order to obtain a robust risk/benefit assessment, future research should sharply focus on patient populations that are most likely to gain from PHBT. This could be prospectively achieved through large multicentre trials. A viable alternative would be including PHBT into large Trauma databases as TARN (The Trauma Audit & Research Network, UK) and the TraumaRegister of the DGU (Deutsche Gesellschaft für Unfallchirurgie, Germany).

PHBT guidelines based, at this stage, only on expert opinion, should be made available to ensure a safe and rational use of this precious resource in the pre-hospital environment.

## Data Availability

The data sets contain individual data allowing to trace responders. We have no permission to share the complete datasests used during this study but they are in an adapted format available from the corresponding author on reasonable request, with identifying data removed.

## Abbreviations

PHBP: Prehospital blood products
PHBT: Prehospital blood transfusion
PRBC: Packed Red Blood Cells
ESA: European Society of Anaesthesiology
SAMU: Service d’Aide Médicale Urgente
FFP: Fresh Frozen Plasma
HEMS: Helicopter Emergency Medical Service
EMS: Emergency Medical Service
NNT: Number Needed to Treat
HR: Hazard Ratio
RCT: Randomised controlled trial
ISS: Injury Severity Score
NISS: New Injury Severity Score
TARN: Trauma Audit and Research Network
DGU: Deutsche Gesellschaft für Unfallchirurgie
DRF: DRF-Luftrettung
ADAC: Allgemeiner Deutscher Automobilclub
EU: European Union
